# Immune Checkpoint Modulators: An Emerging Antiglioma Armamentarium

**DOI:** 10.1155/2016/4683607

**Published:** 2016-01-04

**Authors:** Eileen S. Kim, Jennifer E. Kim, Mira A. Patel, Antonella Mangraviti, Jacob Ruzevick, Michael Lim

**Affiliations:** ^1^Department of Neurosurgery, Johns Hopkins University School of Medicine, Baltimore, MD 21205, USA; ^2^Department of Oncology, Johns Hopkins University School of Medicine, Baltimore, MD 21205, USA

## Abstract

Immune checkpoints have come to the forefront of cancer therapies as a powerful and promising strategy to stimulate antitumor T cell activity. Results from recent preclinical and clinical studies demonstrate how checkpoint inhibition can be utilized to prevent tumor immune evasion and both local and systemic immune suppression. This review encompasses the key immune checkpoints that have been found to play a role in tumorigenesis and, more specifically, gliomagenesis. The review will provide an overview of the existing preclinical and clinical data, antitumor efficacy, and clinical applications for each checkpoint with respect to GBM, as well as a summary of combination therapies with chemotherapy and radiation.

## 1. Introduction

Over the past five years, a series of landmark publications heralded the advances of checkpoint inhibitors as cancer immunotherapy [1–3]. Recent clinical trials have demonstrated significant response rates with anti-CTLA-4 and anti-PD-1 antibodies in patients with late stage melanoma and squamous cell lung cancer [1, 4]. These results, along with the recent FDA approval of ipilimumab (anti-CTLA-4) and nivolumab (anti-PD-1), continue to highlight checkpoint inhibitors' potential as powerful new additions to the modern anticancer armamentarium.

**Figure 1:**
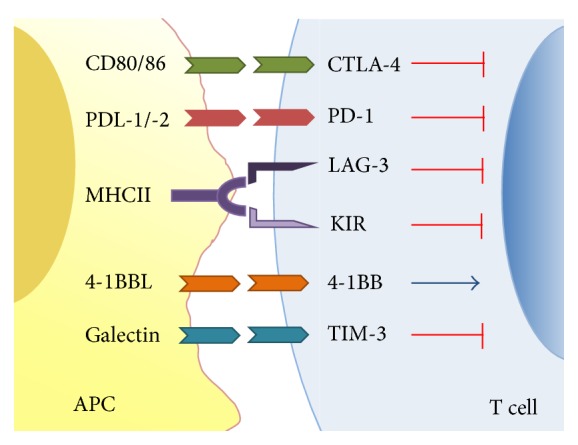
Negative and positive immune checkpoint receptors and ligands.

Preclinical and clinical studies have shown that immunotherapy can improve survival and generate a robust antitumor immune response to improve cancer therapy [5, 6]. Under normal physiologic conditions, immune homeostasis is regulated by a careful balance of activating and inhibitory signals. These “immune checkpoints” (Figure 1) play a critical role in regulating the cells of the immune system. Dysregulation of these checkpoints has been implicated in the pathologically up- or downregulated immune responses seen in chronic infection, autoimmunity, and cancer.

Tumor cells have developed several strategies to exploit these checkpoints and circumvent the host immune defenses. Glioblastoma multiforme (GBM) is the most common central nervous system (CNS) tumor, which has been shown to evade host antitumor response by decreasing immune activation and antigen recognition through several mechanisms. These methods include inducing T cell anergy and lymphopenia, decreasing synthesis of antibodies, increasing immunosuppressive cytokines (i.e., IL10 and TGF-*β*), upregulating inhibitory molecules of T cells (i.e., Fas ligand [FasL] and programmed death ligand-1 [PDL-1]), and recruiting regulatory T cells (Tregs) and myeloid derived suppressor cells (MDSCs) to subdue immune response [7–13].

**Figure 2:**
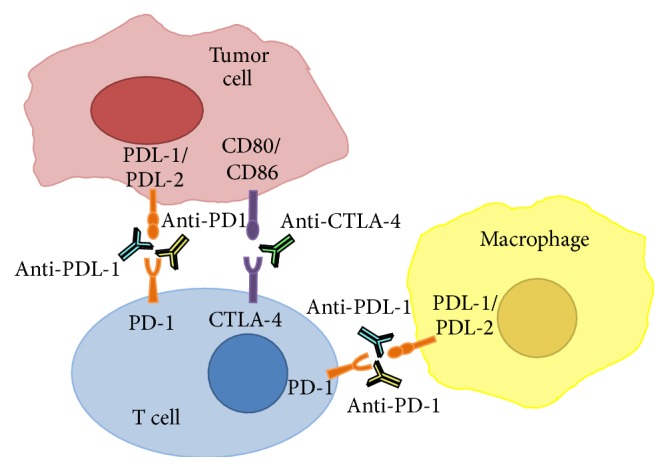
Anti-checkpoint antibodies and their targets.

The recent discovery of lymphatic vessels in the brain has generated much excitement towards an immune approach to treatment of brain malignancies [14]. This finding provides anatomic evidence for immune communications between the periphery and CNS and may support the long-standing theory that activated, circulating T cells can cross the blood brain barrier after peripheral vaccination or checkpoint inhibition. At present, several studies have demonstrated a positive correlation between high lymphocytic infiltration of primary brain tumors and overall survival [15–21]. Targeted immunotherapy has, therefore, emerged as a promising new approach for treatment, based on the principle that augmenting tumor infiltrating lymphocytes (TILs) activity in the tumor microenvironment could translate to tumor regression [22, 23]. Monoclonal antibodies as agonists or antagonists that target checkpoint inhibitors have emerged as potential strategies to restrict TIL inhibiting signals from the tumor and circulating monocytes, block negative signals and cytokines that inhibit T cell activity, and stimulate systemic immunity (Figure 2) [24].

In this review, we will discuss a series of immune checkpoints that have emerged as potential targets for therapeutic blockade, with an emphasis on those pertinent to the treatment of malignant gliomas. This discussion will touch upon cellular mechanisms, clinical relevance, and outcomes of both preclinical and clinical studies pertaining to each checkpoint. We will also address the topics of combination therapy with other checkpoints molecules as well as other modalities.

## 2. Immune Checkpoints

### 2.1. CTLA-4

Cytotoxic T lymphocyte antigen-4 (CTLA-4) is widely regarded as the archetypal T cell intrinsic inhibitory checkpoint. A member of the immune regulatory CD28-B7 immunoglobulin superfamily [25], CTLA-4, acts largely on naïve and resting T lymphocytes to promote immunosuppression through both B7-dependent and B7-independent pathways. The B7-1 (CD80) and B7-2 (CD86) proteins found on the surface of antigen presenting cells (APCs) interact with CD28 receptors on T cells to provide the costimulatory “Signal 2” for T cell activation (“Signal 1” being the primary interaction of the T cell receptor [TCR] and Major Histocompatibility Complex [MHC]). Though the B7:CD28 pathway is one of the best-understood mechanisms for T cell costimulation, it is complicated by the addition of CTLA-4 (CD152), a lymphocyte surface protein with 30% homology to CD28 [26]. This transmembrane glycoprotein is a negative T cell regulator that also associates with B7, but with nearly 20 times greater affinity. CTLA-4:B7 engagement not only is quick and effective, but also segregates and prevents B7 from interacting with the activating CD28 [27–29].

**Figure 3:**
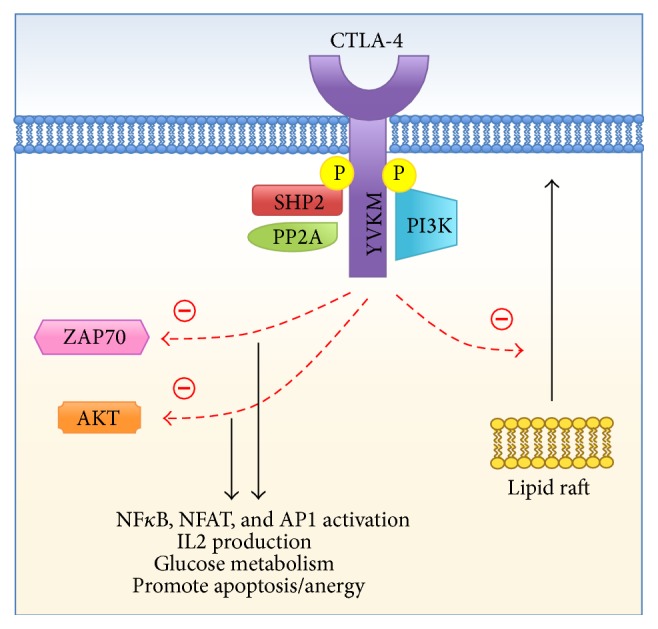
CTLA-4 signaling cascade.

B7-dependent immunosuppression occurs through direct engagement of CTLA-4, which may be expressed in a constitutive or rapidly inducible manner on CD4+, CD8+, and regulatory T cells (Tregs) [30]. Though the exact signaling mechanism for T cell inactivation has not yet been fully characterized, existing evidence suggests that upon phosphorylation, CTLA-4 binds to phosphoinositide 3-kinase (PI3K) via a Tyr-Val-Lys-Met (YVKM) motif and activates phosphatases SHP2 and PP2A. Downstream effects of the proposed signaling cascades (see Figure 3) may include inhibition of metabolism [31, 32], inactivation of transcription factors [33, 34], inhibition of CD28-mediated lipid raft formation, [35, 36], and loss of calcium mobilization required for cell proliferation [37].

As an effector molecule, CTLA-4 modulates the threshold for T cell activation [38]. Along with direct signal transduction, engagement with B7 has been shown to control rapid cell surface accumulation of CTLA-4 [39]. CTLA-4 may also actively capture and remove B7-1 (CD80) and B7-2 (CD86) proteins on the opposing APC through a process of transendocytosis, resulting in “signaling independent” negative T cell regulation [40, 41].

In vivo studies have highlighted the regulatory role that CTLA-4 may play in lymphoproliferation. Early lethality due to uncontrolled polyclonal CD4+ T cell expansion was demonstrated in CTLA-4-deficient mice, ostensibly resulting from dysregulated self-tolerance of peripheral autoantigens [42–44].

#### 2.1.1. Preclinical Evidence

The role of CTLA-4 in glioma maintenance is complex and incompletely understood. While early characterizations of glioma tissue noted dramatic CD4+ lymphopenia and T cell anergy [45–47], the mechanisms by which gliomas achieved global immunocompromise were not yet known. Studies from the early 2000s implicated CTLA-4 in the development of Tregs, a population of immune suppressor cells that is often expanded in gastric [48, 49], pancreatic [50, 51], ovarian [52], and lung cancers [52]. In 2006, using flow cytometry on human GBM samples, El Andaloussi and Lesniak demonstrated that the number of FOXP3+ Tregs were significantly increased in TIL populations compared to controls and that CTLA-4 expression was also elevated within the glioma Treg population compared to those in the control samples [8]. That same year, Fecci et al. reported their findings that while absolute CD4+ cell counts (including CD4+ T helper cells and CD4+CD25+FoxP3+CD4RO+ Tregs) were lower in malignant glioma samples compared to controls, Tregs represented an increased fraction of the existing T cells, and though diminished in number, they were sufficient to significantly impair immune responsiveness [53]. These findings have helped implicate CTLA-4 in the maintenance of an immunosuppressive tumor microenvironment and highlight its potential as a target for immunotherapy in malignant gliomas.

In a follow-up study by Fecci et al, monoclonal anti-CTLA-4 antibody was administered in murine glioma-bearing mice to investigate the immune consequences of CTLA-4 checkpoint blockade. A long-term survival of 80% was reported in the treated group, as well as a restoration of CD4+ proliferation and antitumor capacity. Interestingly, the treatment effects seemed to be exclusive to the CD4+ helper T cell compartment, while Tregs remained functionally unaffected or unsuppressed [9]. Additional animal studies explored the effects of combining anti-CTLA-4 with other immunotherapies. Grauer et al. reported a 50% survival with anti-CTLA-4 alone, compared to 100% survival in mice treated with both anti-CTLA-4 and anti-CD25 (alpha chain of the IL-2 receptor) [54]. Agarwalla et al. found that while high dose anti-CTLA-4 alone was ineffective against large, well-established tumors, the addition of a whole tumor cell vaccination (Gvax) significantly improved long term survival in mice with murine intracranial gliomas [55]. Findings such as these have helped promote the development of clinical trials using anti-CTLA antibody for malignant gliomas.

#### 2.1.2. Clinical Evidence

In light of promising results in animal models, clinical testing of two fully humanized anti-CTLA-4 antibodies, ipilimumab (Bristol Meyer-Squibb) and tremelimumab (Pfizer), began in 2000. The findings from subsequent studies culminated in the 2011 FDA approval of ipilimumab for the treatment of unresectable or metastatic melanoma [3, 6, 41, 56]. With regard to GBM, The National Cancer Institute has begun a Phase I trial to identify safety and dosage of ipilimumab and/or nivolumab with temozolomide in newly diagnosed glioblastoma (NCT02311920). In addition, a randomized, 2-arm, Phase II-III study of ipilimumab in combination with standard-of-care temozolomide for the treatment of newly diagnosed glioblastoma is also currently underway, helmed by the Radiation Therapy Oncology Group (RTOG 1125) [57].

### 2.2. PD-1/PDL-1

Like CTLA-4, programmed cell death protein 1 (PD-1, also known as CD279) is an inhibitory receptor that negatively regulates the immune system. However, while CTLA-4 mainly affects naïve T cells, PD-1 is more broadly expressed on immune cells and regulates mature T cell activity in peripheral tissues and in the tumor microenvironment [41].

The PD-1 receptor binds two ligands, PD ligand 1 (PDL-1, also known as B7-H1 or CD274) and PDL2 (B7-DC or 273) [58–61], each belonging to the same B7 family as the B7-1 and B7-2 proteins that interact with CD28 and CTLA-4. In the first paper detailing the discovery of the ligand, Dong et al. noted that ligation of PDL-1 not only decreased IFN*γ*, TNF*α*, and IL-2 production but also stimulated production of IL10, an anti-inflammatory cytokine associated with decreased T cell reactivity and proliferation as well as antigen-specific T cell anergy [58, 60, 61]. PDL2 ligation also results in T cell suppression, but where PDL-1-PD-1 interactions inhibits proliferation via cell cycle arrest in the G1/G2 phase [62], PDL2-PD-1 engagement has been shown to inhibit TCR-mediated signaling by blocking B7:CD28 signals at low antigen concentrations and reducing cytokine production at high antigen concentrations [59].

**Figure 4:**
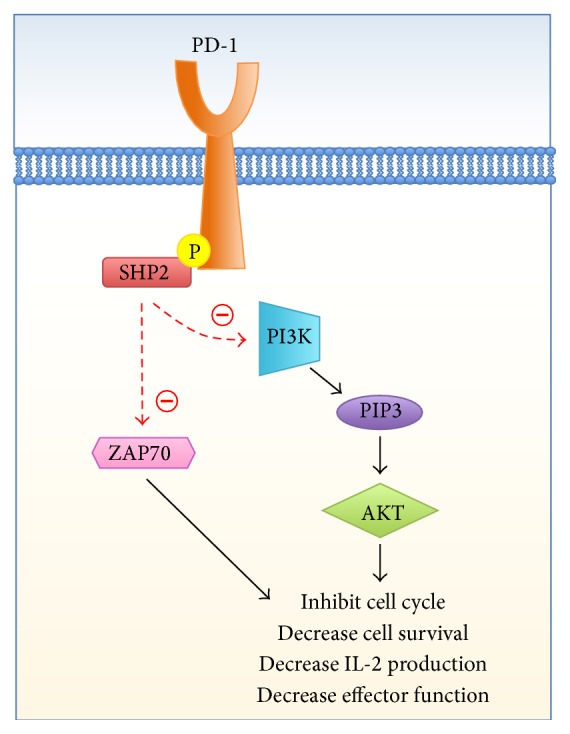
PD-1 signaling cascade.

Though both CTLA-4 activity and PD-1 activity have immunosuppressive effects, PD-1 relies on different signaling pathways and mechanisms to suppress the T cell inflammatory response and limit autoimmunity (Figure 4).

Ligation of this 288-amino acid transmembrane receptor results in the dephosphorylation (and deactivation) of ZAP70 and the recruitment of SHP2. Upon binding PD-1, SHP-2 directly dephosphorylates PI3K, which inhibits downstream activation of Akt and thereby decreases production of inflammatory cytokine production and cell survival proteins (i.e., Bcl-xL) [63, 64]. Of note, PD-1 activity may be countered or overcome by strong TCR signaling or concomitant CD28 [65] or IL-2 [66] costimulation, allowing recovery of cytokine production and cell survival [61].

#### 2.2.1. Preclinical Evidence

PDL-1 has been shown to be highly expressed on multiple malignant gliomas, as compared to normal brain or benign tumor tissues [67–70]. The mechanism for ligand upregulation has been elucidated in part by Parsa et al., who found that loss of the phosphatase and tensin homolog (PTEN) led to increased PDL-1 gene transcription; furthermore, gliomas with wild-type PTEN were more likely to be lysed by tumor-specific T cells than gliomas with mutant or inactivated PTEN [68]. The presence of PDL-1 has been associated with potent inhibition of CD4+ and CD8+ T cell activation and cytokine release (IFN*γ*, IL2, and IL10) [67]. PDL-1 expression levels have also been shown to have significant correlation with tumor grade [71]. Using a mouse orthotopic glioblastoma model, Zeng et al. demonstrated that the combined used of anti-PD-1 and focal radiation therapy led to robust antitumor activity and immunologic memory, as demonstrated by significantly improved survival, increased tumor infiltration of CD8+ T cells, and decreased Tregs populations [5]. These findings have spurred interest in further testing of PD-1 blockade in the clinical trials setting.

#### 2.2.2. Clinical Evidence

At present, several forms of monoclonal anti-PD-1 and anti-PDL-1 antibodies are undergoing clinical development, several of which have shown promising results in early Phase I and II trials (Table 1).

Therapeutic IgGs that target the PD-1 receptor include AMP-224 (Amplimmune), Pembrolizumab (Merck), Nivolumab (BMS), and Pidilizumab (CureTech). Human IgGs targeting the PDL-1 ligand include BMS-936559 (BMS), MEDI4736 (Medimmune), MPDL3280A (Genentech), and MSB0010718C (Merck); additionally, rHigM12B7 (Mayo Foundation) is a human IgM that targets the PDL2 ligand.

Recent results from a clinical trial examining the safety and efficacy of Nivolumab with and without ipilimumab have shown that monotherapy with Nivolumab had fewer treatment related adverse effects than combination therapy and that immune therapy seems to have biologic effects. This has led to Phase III of the trial comparing the safety and efficacy of Nivolumab versus Bevacizumab with or without ipilimumab (NCT02017717). There are several clinical trials recruiting patients to study the effects of anti-PD-1 in patients with GBM. These trials include a Phase I/II clinical trial (NCT01952769) to study the safety and efficacy of Pidilizumab in diffuse intrinsic pontine glioma and relapsed GBM, a Phase II trial of neoadjuvant Nivolumab in primary and recurrent GBM (NCT02550249), a Phase II trial of Pembrolizumab in recurrent GBM (NCT02337686), and several trials examining the effects of combination therapy of anti-PD-1 antibodies with Temozolomide with and without radiation therapy (NCT02311920, NCT02530502), INCB24360 (NCT02327078), FPA008 (NCT02526017), and dendritic cell vaccine (NCT02529072).

## 3. Additional Checkpoints

### 3.1. LAG-3

Lymphocyte-activation gene 3 (LAG-3, also known as CD223) is a CD4-related transmembrane protein that competitively binds MHC II and acts as a coinhibitory checkpoint for T cell activation [72, 73]. The mechanism by which LAG-3 negatively regulates the TCR-CD3 complex and inhibits T cell proliferation and cytokine production is not well understood, but several studies have suggested that the inhibitory function depends on a conserved KIEELE motif in the protein's cytoplasmic domain [72–74]. An additional domain binds LAP (LAG-3-associated protein), which may play a role in microtubule association after TCR engagement [75].

LAG-3 is expressed in vivo on the surface of activated CD4+, CD8+, and NK cells [75, 76] under inflammatory conditions. In vitro studies have shown that LAG-3 is upregulated by IL12 and promotes the production of IFN*γ* [77]. LAG-3 expression is required for maximal Treg function, and ectopic expression may be sufficient for inducing regulatory activity, with suppressive capacities comparable to ectopically expressed FOXP3 [78, 79]. LAG-3 may also play a role in regulating DC function; engagement with DC MHCII molecules has been shown to induce morphologic changes and upregulate IL12 and TNF*α* secretion [76]. In a study by Workman and Vignali, LAG-3(−/−) T cells exhibited the following characteristics as compared to LAG-3+ cells: (1) delayed cell cycle arrest after stimulation with a superantigen, (2) greater proliferation after in vivo stimulation, (3) and higher numbers of memory T cells after viral exposure [73]. These data suggested LAG-3 plays an important role in regulating T cell expansion, a hypothesis that was further supported by a study by Huang et al. Using LAG-3 knockout mice, the authors demonstrated that, compared to wild-type Tregs, more than double the number of LAG-3(−/−) Tregs were required to control CD4+ helper T cell proliferation at high antigen peptide concentrations; furthermore, the authors reported that administration of anti-LAG-3 antibodies resulted in a reversal of Treg-mediated immune suppression [78]. Grosso et al. also employed antibodies against LAG-3 to increase proliferation and effector function of tumor-specific CD8+ cytotoxic T cells and resulting in disrupted tumor architecture and growth inhibition [80]. A recent study by Woo et al. demonstrated the efficacy of combined checkpoint blockade using three distinct tumor types (B16 melanoma, MC38 colorectal adenocarcinoma, and Sa1N fibrosarcoma); in each of these tumors types, tolerized T cells were found to coexpress LAG-3 and PD-1. Whereas treatment with anti-LAG-3 alone or anti-PD-1 alone delayed tumor growth in a minority of treated mice (0–40%), dual therapy with anti-LAG-3 and anti-PD-1 resulted in complete tumor regression in 70 and 80% of mice with fibrosarcoma and colorectal tumors, respectively. Though no therapeutic effects were observed in the melanoma-inoculated mice, these findings provided compelling evidence for a synergistic benefit of combination checkpoint blockade [81]

### 3.2. TIM-3

T cell immunoglobulin mucin 3 (TIM-3) was discovered in 2002 as a marker of IFN*γ* producing CD4+ and CD8+ T cells in mice and humans [82, 83]. A type I glycoprotein receptor that binds to S-type lectin galectin-9 (Gal-9), TIM-3, is a widely expressed ligand on lymphocytes, liver, small intestine, thymus, kidney, spleen, lung, muscle, reticulocytes, and brain tissue [84]. Binding of Gal-9 by the TIM-3 receptor triggers downstream signaling to negatively regulate T cell survival and function. In vitro studies have shown that Gal-9 induced TIM-3 activation induced intracellular calcium influx, aggregation, and cell death (mixed apoptosis and necrosis) of CD4+ T cells; additionally, Gal-9 administration in vivo can cause rapid elimination of IFN*γ*-producing CD4+ T cells and suppress Th1-mediated autoimmunity [85].

TIM-3 is a marker of CD8+ T cell exhaustion in the setting of chronic viral infections and immunogenic tumor microenvironments [82, 86–90]. TIM-3+PD-1+ TILs have been identified in murine models of colon adenocarcinoma, breast adenocarcinoma, and melanoma; coexpression of these two T cell “exhaustion” markers has been shown to be the most functionally impaired group of CD8+ TIL populations as determined by lowest IL2, TNF and IFN*γ* production and progression through the cell cycle [90, 91]. In advanced AML tumor models where PD-1+TIM-3+ CD8+ cells have been correlated with disease progression, dual therapy with anti-PDL-1 and TIM-3Ig has been shown to significantly decrease tumor burden and improve survival [86].

Recent evidence suggests that TIM-3 may also play a role in myeloid-derived suppressor cell (MDSC) development. Composed of a heterogeneous group of CD11b+Gr1+ myeloid cells, MDSCs are powerful T cell suppressors that have been shown to proliferate under conditions of infection, autoimmunity, trauma, and malignancy, and their presence has been identified as negative predictive factor predictor for oncologic outcomes [10]. Both Gal-9 and transgenic TIM-3 overexpression have been shown to induce MDSC expansion, with subsequent T cell inhibition [92]; conversely, tumor growth was found to be significantly delayed in TIM-3(−/−) mice implanted with T1 mammary adenocarcinoma, as compared to TIM-3+ wild type mice [92].

### 3.3. KIR

Killer immunoglobulin-like receptors (KIRs) comprise a diverse repertoire of MHCI binding molecules that negatively regulate NK function to protect cells from NK-mediated cell lysis. KIRs are generally expressed on NK cells but have also been detected on tumor specific CTLs [93]. Members of the KIR family of molecules contain 2-3 Ig ectodomains and cytoplasmic tails of variable length [94]. While some “noninhibitory” KIRs have truncated cytoplasmic tails, others possess longer tails containing two immune receptor tyrosine-based inhibitory motifs (ITIMs) that mediate downstream signaling and confer anti-NK potential [95–98]. The KIR locus is most likely polymorphic and polygenic, with inhibitory KIR haplotypes remaining relatively specific for HLA-B and HLA-C ligands, while noninhibitory phenotypes display greater variability [99].

Unlike adaptive B and T cells, NK cells lack such meticulous antigen sensitivity and instead rely on several activating and inhibitory receptors to modulate and direct their killing capacity [100]. When expressed on the cell surface, KIRs may play a role in inducing NK tolerance through a process of “licensing,” in which each inhibitory receptor recognizes a self HLA class I molecule and prevents NK activation against autoantigens and self-tissue [101, 102]. Knowledge of these germline-encoded receptors has provided valuable insight into the mechanisms of NK-tumor interactions [103, 104]. The phenomenon of NK-dependent rejection of syngeneic or human solid and hematopoietic tumor grafts [105, 106] is partially explained by the “missing” self-recognition phenomenon, where NK cells have been found to target aberrant cells that specifically lack self MHC I expression [107–109]. Though controversial, a few studies have also demonstrated that a lack of KIR ligands or KIR ligand incompatibility with foreign tissues is associated with improved survival and lower relapse rates [110–112] and suggest KIR inhibition as a viable means of enabling or augmenting NK cell-mediated antitumor lytic activity. This hypothesis has been borne out in adoptive transfer experiments of KIR-ligand mismatched or KIR-ligand nonexpressing NK cells which led to significantly increased cytotoxicity of multiple tumor cell lines [113, 114]. KIR blockade using anti-KIR antibodies has also been shown to prevent tolerogenicity and reconstitute NK-mediated cell lysis in both in vivo and in vitro hematopoietic cancer models [115–117].

### 3.4. 41BB

A member of the tumor necrosis factor (TNF) receptor superfamily that includes the FAS receptor (apoptosis antigen), CD40 (T cell costimulatory receptor), CD27 (TNF receptor), and CD30 (tumor marker), and 4-1BB (CD137) is a Type II transmembrane glycoprotein [118] that is inducibly expressed on primed CD4+ and CD8+ T cells [119], activated NK cells, DCs, and neutrophils [120] and acts as a T cell costimulatory molecule when bound to the 4-1BB ligand (4-1BBL) found on activated macrophages, B cells, and DCs [121, 122]. Ligation of the 4-1BB receptor leads to activation of the NF-*κ*B, c-Jun and p38 signaling pathways [123] and has been shown to promote survival of CD8+ T cells, specifically, by upregulating expression of the antiapoptotic genes BcL-x(L) and Bfl-1 [124]. In this manner, 4-1BB serves to boost or even salvage a suboptimal immune response [120]. Its expression may also be contingent on activation of the B7:CD28 pathway (see above section on* CTLA-4*), with 4-1BB producing its own feedforward loop to maintain T cell activity, and the B7-CD28 complex serving to temper the immune response and protect against inappropriate immune activation [21].

Unlike negative T cell regulators (i.e., CTLA-4, PD-1, LAG-3, and TIM-3), 4-1BB is an activating checkpoint that mediates prosurvival and proinflammatory signaling pathways. 4-1BB costimulation has been shown to profoundly enhance antigen-specific CD8 T cell survival and proliferation [125] and has therefore become a target of interest in tumor immunotherapy, especially against poorly immunogenic tumors for which the host antitumor immune response may prove inadequate. Monoclonal agonist antibodies are one promising method of harnessing the proinflammatory potential of this checkpoint molecule. Anti-4-1BB antibodies have been shown to cause tumor regression in animal models of sarcoma and mastocytoma [119], breast cancer [126], and metastatic colon carcinoma [127] with concomitant increase in tumor selective cytotoxic T cell activity. Synergy with IL-12 gene therapy and anti-4-1BB antibody [127] or local 4-1BB gene [126] delivery has also been shown with significant tumor rejection and long-term immunity seen in metastatic breast and colon cancer models. In intracranial tumor models, anti-41BB has been shown to have moderate cure rates (2/5 mice with GL261 glioma and 4/5 with MCA205 sarcoma), but no effect against the poorly immunogenic B16/D5 melanoma model [128]. Adoptive transfer experiments have also been used to highlight 4-1BB's role in antitumor immunity. CD28 and 4-1BB costimulated T cells adoptively transferred into mice bearing poorly immunogenic melanoma have been shown to result in a 60% cure rate [129] and prolong survival in murine fibrosarcoma models [130]. Whole cell vaccines using tumor cells transfected with 4-1BBL cDNA have also been shown to induce vigorous antitumor CD8+ T cell activity and long term survival in various tumor models [131–134]. However, the technical difficulty and feasibility of culturing and administering lymphocyte or transfected tumor cells for either adoptive transfer or whole cell vaccination have limited their translation into clinical practice.

### 3.5. GITR

Glucocorticoid-induced TNFR family related gene (GITR) is a member of the tumor necrosis factor receptor (TNFR) superfamily that is constitutively or conditionally expressed on Treg, CD4, and CD8 T cells [135, 136]. Initially described as a unique CD4+CD25+FoxP3+ Treg marker [137], subsequent studies demonstrated rapid upregulation of GITR on effector T cells following TCR ligation and activation [138–142]. The human GITR ligand (GITRL) is constitutively expressed on APCs in secondary lymphoid organs and has also been found on nonlymphoid tissues including vascular endothelial and various epithelial cells [135, 143]. The downstream effect of GITR:GITRL interaction is believed to be at least twofold, including (1) attenuation of Treg activity and (2) enhancement of CD4+ T cell activity [137–139, 141, 144, 145]. The net result is a reversal of Treg-mediated immunosuppression and increased immune stimulation [142, 146].

Like the 4-1BB costimulatory molecule, GITR is an activating checkpoint that enhances inflammatory pathways and host immune response. Overexpression or experimental GITR agonism is associated with autoimmunity [138, 140, 147] and pathologic inflammatory responses such as in asthma [148] and post-stroke states [149]. Preclinical studies have elucidated the differential effects of GITR upregulation on Tregs versus effector T lymphocytes, and its potential role in facilitating the antitumor immune response. Using anti-GITR monoclonal antibodies, Cohen et al. demonstrated that GITR agonism led to lower intratumoral Treg accumulation, loss of FoxP3 expression, decreased Treg suppressor function, and, ultimately, regression of B16 melanoma in mouse models [150]. While these findings were initially implicated Tregs as the primary substrate for GITR:GITRL interactions, subsequent studies have suggested that effector T cells, as opposed to Tregs, may be the principal mediators of the GITR signaling pathway [139–141]. Using GITR knockout mice that still retained functional Treg populations, Stephens et al. elegantly demonstrated that GITR engagement on CD4+CD25− T cells, and not CD25+ Treg cells, was required to abrogate Treg suppressive activity [151]. Conversely, antagonizing GITRL using blocking antibodies seemed to increase CD4+ T cell susceptibility to Treg-mediated suppression [151]. Additional studies that demonstrated the efficacy of anti-GITR agonist antibodies in inducing tumor regression and preventing regrowth upon secondary challenge have raised interest in GITR as a potential target of tumor immunotherapy [138, 152, 153].

#### 3.5.1. Clinical Evidence

At present, there are no clinical trials for GBM involving IMP321 (a soluble LAG-3 chimeric IgG1 and MHCII agonist), anti-TIM-3 antibody, IPH2101 (anti-KIR), BMS-663513 (a fully humanized anti-4-1BB agonist antibody), or TRX518 (a first in class, humanized anti-GITR monoclonal antibody). However, these immune modulators have tremendous therapeutic potential for the treatment of CNS tumors.

## 4. Integrating Checkpoint Inhibitors into the Standard of Care

Despite aggressive treatment with chemotherapy and radiation, the refractory nature of high-grade gliomas has become strong motivation to seek novel treatment regimens. The clinical successes of immunomodulating antibodies in both CNS and non-CNS cancers have raised the possibility of adding checkpoint inhibitors to the current anticancer armamentarium as a complementary or even synergistic modality.

Unlike vaccine therapies or adoptive cell transfer, checkpoint inhibition is a nonspecific strategy that relies on generalized activation of the immune system. While T cells are the best-characterized targets of checkpoint inhibition at present, it is becoming clear that these therapies have wide-ranging effects on other immune players such as NK cells, monocytes, macrophages, and dendritic cells [78, 100, 154, 155] (Figure 2). Nonspecific checkpoint-based therapies may therefore benefit from concurrent therapies that either deplete immunosuppressive cells (i.e., chemotherapy) or increase access to tumor-specific antigens (i.e., ionizing radiation).

The following discussion will focus on the possible synergistic effects of concurrent chemoradiation therapy and the challenges of integrating checkpoint inhibitors into the current standard of care.

### 4.1. Checkpoint Inhibitors and Radiation Therapy

RT is a nonselective cytocidal treatment modality that targets rapidly dividing cells. T cells, which are the main effectors of cancer immunotherapy, are known to be exquisitely sensitive to its effects [156, 157]. Studies testing combined RT and TMZ [158] or RT and steroid [47] regimens have demonstrated significant, long-lasting drops in CD4 counts with concomitant systemic immune compromise. Though these findings could suggest an antagonistic interaction between RT and immunotherapy, the significant cellular and stromal destruction caused by ionizing radiation has been shown to act as a powerful “danger,” or activation, signal to the host immune system [159, 160]. Apoptotic tumor cells provide APCs with tumor-specific antigens that can be presented on MHC class I molecules to CD8+ cells, leading to enhanced, antitumor immune activation [161–163]. RT has also been shown to counteract MHC downregulation, a strategy used by GBM to escape immune detection [164, 165]; a study by Newcomb et al. reported a significant upregulation of the *β*2-microglobulin light chain subunit of the MHCI molecule in GL261 glioma cells following whole body radiation therapy [166].

Elucidating the pathways for radiation-induced immune stimulation provides a mechanism for the observed synergy between radiation and immunotherapy. Prolonged survival with the addition of anti-CTLA-4 to stereotactic radiosurgery has been reported in breast cancer-bearing mice, largely attributed to CD8+ T cell activity [167]. Although it has not been seen in GBM, combination therapy with ipilimumab (anti-CTLA-4 antibody) and local radiation has also been shown to cause tumor regression at both irradiated and nonirradiated sites—the latter known as the abscopal effect [168, 169]. Zeng et al. demonstrated that the addition of SRS to PD-1 blockade increased in vitro expression of proinflammatory molecules such as MHCI, CXCL16, and ICAM and correlated with a survival advantage in glioma-bearing mice [5]. The results of these preclinical studies indicate that RT can work synergistically with checkpoint inhibitors, and at present, a Phase I trial is underway testing the combined used of Pembrolizumab and radiation in GBM (NCT02530502). Results from these studies will help guide future strategies to integrate immunotherapy into the current standard of care therapeutic regimen.

### 4.2. Checkpoint Inhibitors and Chemotherapy

Approved by the FDA in 2001 for refractory anaplastic astrocytomas and in 2005 for newly diagnosed GBMs, TMZ is a second-generation DNA alkylating agent that is currently the chemotherapeutic standard for the treatment of malignant gliomas. Since its adoption as a first-line agent, population studies have demonstrated an increase in 2-year survival from 7% in cases that were diagnosed between 1993 and 1995 to 17% in those diagnosed between 2005 and 2007 [170]. Use of TMZ in combination with radiation has also been shown to increase two-year survival from 10.4% to 26.5%, as compared to radiation monotherapy [171].

Chemotherapy has been widely hypothesized to be antagonistic or counterproductive to immunotherapy due to its systemic immune toxic effects. Cytotoxic drugs such as TMZ have been associated with severe lymphopenia [172, 173]. In a prospective, multicenter study of patients with high-grade gliomas, Grossman et al. observed long-lasting, systemic CD4+ lymphodepletion with poor clinical outcomes in patients who underwent treatment with oral TMZ and radiation. In this study, median CD4 count was 664 cells/mm^3^ before treatment, reached its lowest point at 255 cells/mm^3^ two months after the start of TMZ + RT, and remained persistently low for the duration of observation (12 months) [158]. In theory, these effects—in combination with the locally immunosuppressive tumor microenvironment—could abrogate immunotherapy's efficacy by depleting the peripheral pool of effector T cells.

Contrary to these suppositions, numerous clinical studies combining chemotherapy with immunotherapy such as monoclonal antibodies, active specific immunotherapy, and adoptive lymphocyte immunotherapy have shown promising results, though larger studies are needed to verify and assess efficacy [174]. Heimberger et al. published a case study in 2008 demonstrating successful immune activation in a GBM patient following treatment with both TMZ and EGFRvIII vaccine [7]. Of note, the authors observed no significant decline in CD4+ and CD8+ T cell counts and concluded that as long as the cytotoxic chemotherapy was administered outside of the vaccine's therapeutic window, the two modalities could be used in a synergistic manner [7]. Furthermore, some authors have suggested the use of local or intratumoral TMZ as a less immunosuppressive alternative compared to oral TMZ. Using glioma-bearing mice, Brem et al. found that polymeric implants for local TMZ delivery were associated with improved survival, and that the addition of RT prolonged survival even further without additional toxicity [175]. Fritzell et al. later demonstrated that intratumoral TMZ may synergistically increase survival rates in immunized mice by sustained proliferation of CD8+ T cells and decreased intratumoral immunosuppressive cells such as myeloid-derived suppressor cells (MDSCs) [176].

With respect to checkpoint inhibitors, these findings imply that carefully timed, interdigitated or alternating chemotherapy would not only protect immunotherapy-activated effector T cells but also ablates immunosuppressive Tregs that could otherwise reduce the efficacy of immunomodulating antibodies [7, 177]. The use of intratumoral chemotherapy may also further protect the effector T cells and provide a survival advantage due to a more robust immune profile. At present, there are no published clinical trials data on the use of TMZ plus checkpoint inhibitors. Further preclinical and clinical studies will be required to examine the risks and benefits of this particular multimodal therapeutic strategy.

## 5. Summary

Immune checkpoint therapy has emerged as a welcome and potent addition to the current arsenal of anticancer treatment. While certain checkpoint blockades such as CTLA-4 and PD-1 have proven clinically successful, both alone and in conjunction with each other, there are several other targets that such as LAG-3, TIM-3, KIR, and GITR that have shown promise for passive immunotherapy. Anti-CTLA-4 and anti-PD-1 have had promising outcomes in preclinical studies for the treatment of malignant GBMs. Those studies have spurred further ongoing clinical trials that look to solidify immune therapy as a mainstay for treating primary and recurrent brain tumors. Checkpoint inhibitors may be effective not only as monotherapy, but also in combination with chemotherapy and/or radiation therapy. Synergy between the antibodies and either of the two conventional modalities could lead to significant improvements in tumor regression and overall survival. Further research on the mechanisms and therapeutic efficacy of specific antibodies, as well as their interactions with other treatment modalities, is needed to successfully incorporate checkpoint modulators into the current standard of care.

## Figures and Tables

**Table 1:** Immune checkpoint antibodies under clinical development.

Target	Biological function	Agent	Stage of clinical development
CTLA-4	Inhibitory receptor	Ipilimumab Tremelimumab	Phase I/II/III/IV Phase I/II/III

PD-1	Inhibitory receptor	Nivolumab (MDX1106, BMS-936558) Pembrolizumab (MK-3475) Pidilizumab (CT-011)	Phase I/II/III/IV Phase I/II/III Phase I/II

PD-L1	Ligand for PD-1	BMS935559 (MDX1105) MPDL3280A MEDI4736 MSB0010718C	Phase I Phase I Phase I Phase I

PD-1-positive T cells	PD-1 inhibitor	AMP-224	Phase I

LAG-3	Inhibitory protein	IMP321	Phase I/II (terminated)

KIR	Inhibitory receptor	Lirilumab (IPH2101, BMS)	Phase I/II

4-1BB	Stimulatory receptor	Urelumab (BMS-663513)	Phase I

GITR	Stimulatory receptor	TRX518	Phase I

TIM-3	Inhibitory receptor	Anti-TIM-3	Preclinical
